# Host intestinal biomarker identification in a gut leakage model in broilers

**DOI:** 10.1186/s13567-019-0663-x

**Published:** 2019-06-18

**Authors:** Fien De Meyer, Venessa Eeckhaut, Richard Ducatelle, Maarten Dhaenens, Simon Daled, Annelike Dedeurwaerder, Maarten De Gussem, Freddy Haesebrouck, Dieter Deforce, Filip Van Immerseel

**Affiliations:** 1 Department of Pathology, Bacteriology and Avian Diseases, Faculty of Veterinary Medicine, Ghent University, Salisburylaan 133, 9820 Merelbeke, Belgium; 2 Laboratory for Pharmaceutical Biotechnology, Faculty of Pharmaceutical Sciences, Ghent University, Ottergemsesteenweg 460, 9000 Ghent, Belgium; 3 Poulpharm BVBA, Prins Albertlaan 112, 8870 Izegem, Belgium; 4 Vetworks BVBA, Knokstraat 38, 9880 Poeke, Belgium

## Abstract

Intestinal health problems are a major issue in the poultry industry. Quantifiable easy-to-measure biomarkers for intestinal health would be of great value to monitor subclinical intestinal entities that cause performance problems and to evaluate control methods for intestinal health. The aim of the study was to identify host protein biomarkers for intestinal inflammation and intestinal barrier damage. Proteomic analysis was conducted on ileal and colonic content samples of broilers under an experimental gut damage and inflammation model. Effects of the challenge treatment resulted in a worse gut condition based on macroscopic gut appearance (*p *< 0.0001). Also microscopic changes such as shortening of the villi and increased crypt depth (*p *< 0.0001) as well as higher infiltration of T-lymphocytes (*p* < 0.0001) were seen in the duodenal tissue of challenged animals. Several candidate proteins associated with inflammation, serum leakage and/or tissue damage were identified with an increased abundance in intestinal content of challenged animals (*p *< 0.05). Conversely, brush border enzymes were less abundant in intestinal content of challenged animals (*p *< 0.05). These candidate biomarkers have potential to be used in the field for detection of gut barrier failure in broilers.

## Introduction

The production and consumption of broiler meat is rising rapidly worldwide [1, 2]. Of all terrestrial meat-producing animals, broilers probably have the highest relative daily weight gain and the lowest feed conversion. Therefore, broiler meat is considered to be a relatively sustainable source of animal protein [3]. Unfortunately, disturbances of intestinal tract function are common in broilers, which can hamper their feed conversion due to inefficient digestion and absorption of nutrients [4]. These disturbances are associated with necrotic enteritis, coccidiosis [5, 6] and a range of ill-defined enteric syndromes of unclear etiology, sometimes classified under the common denominator of “dysbiosis”. They constitute the major challenge to the broiler production today.

The intestinal mucosa is responsible for absorption of nutrients. To maintain a good intestinal barrier function, an optimal balance between the mucus layer, the intestinal epithelium and the microbiota is crucial [7]. A single layer of columnar epithelial cells which are connected by tight junctions is a crucial component of the intestinal barrier. It acts as a first protection against invasion of potentially harmful microorganisms, antigens and toxins from the intestinal lumen [8, 9]. Reduced tight junction integrity and epithelial damage can thus result in a ‘leaky gut’ and are likely the main drivers for gut wall morphology changes, inflammation, systemic infection and malabsorption [10, 11]. Poor gut health in broilers has indeed been associated with intestinal villus shortening [12], immune cell infiltration in the mucosal wall [13], and systemic spread of bacteria to organs, as is the case in bacterial chondronecrosis with osteomyelitis in broilers [14]. The importance of intestinal health in broilers is illustrated by the observation that more than 50% of the therapeutic use of antibiotics in broilers in the European Union, is to control intestinal pathologies [15]. Therapeutic usage of antibiotics may be the sole viable solution in flocks with severe advanced intestinal health problems.

Alternative, non-antibiotic, solutions for maintaining intestinal health include diet optimization and the use of feed additives, such as probiotics, acidifiers and essential oils [16]. The use of these products as preventive or curative tool requires early detection of intestinal health problems in broilers. Unfortunately, for the early stages of intestinal health defects, clinical indications are lacking. For measurement of the subclinical level of dysbiosis, field veterinarians and researchers use a macroscopic scoring system [17] but this is labor-intensive and requires necropsy of the animals. These observations have fueled intensive research hunting for specific biomarkers of intestinal health, or deficiency thereof [18]. In humans, fecal markers such as calprotectin, a stable protein that is contained in secretory granules of neutrophils, are used to evaluate inflammation in case of severe gut disease [19, 20]. In poultry, several attempts have been reported to identify suitable biomarkers for gut barrier failure, mostly in serum samples, either with or without an experimental intervention [10, 21]. Most of these studies have analyzed specific candidate biomarkers, often homologues of molecules considered to be of value in detection of gut health problems in mammals. Until now, few seem to be ready for practical use in the field. Recently, ovotransferrin has been proposed as fecal biomarker for intestinal leakage caused by coccidiosis and necrotic enteritis in broilers [22].

A first step in the development of a reliable, rapid and non-invasive diagnostic assay to monitor gut health is the identification of suitable biomarkers. Biomarkers for intestinal health defects, that can be used on fresh fecal droppings or litter, would be a huge asset. The aim of this study, therefore, was to identify intestinal biomarkers for evaluation of intestinal health using a gut damage model in broilers. Preferably the biomarkers should be of peptide or protein nature, as this may ultimately allow detection by a simple immunoassay. Therefore, an unbiased proteomic approach was used.

## Materials and methods

### Animal trials and sample collection

The experiment was approved by the Ethical Committee of Poulpharm BVBA, Izegem, Belgium (bacterial enteritis trial; P1606-FP) with approval number LA1400564. The animal experiment was carried out in accordance with approved guidelines.

### Bacterial inoculum

A bacterial inoculum consisting of a mixture of *Escherichia coli* (G.78.71), *Enterococcus faecalis* (G.78.62), *Lactobacillus salivarius* (LMG22873), *Lactobacillus crispatus* (LMG49479), *Clostridium perfringens* (netB-) (D.39.61) and *Ruminococcus gnavus* (LMG27713) was prepared. Luria–Bertani broth (LB, Oxoid) was used for growing *E. coli*. *E. faecalis* and *C. perfringens* were grown in Brain Heart Infusion (BHI, Sigma, Belgium) broth. Man–Rogosa–Sharpe (MRS, Oxoid) medium was used for the growth of *L. crispatus* and *L. salivarius*. For the growth of *R. gnavus*, anaerobic M2GSC medium (pH 6) as described by Miyazaki et al. [23] was used but with 15% clarified rumen fluid instead of 30% and addition of 1 mg/mL cysteine HCl and 4 mg/mL NaHCO_3_ after autoclaving. *E. coli* and *E. faecalis* were cultured in aerobic conditions at 37 °C. *Lactobacillus* spp. were cultured in an micro aerobic (5% O_2_) incubator, *C*. *perfringens* and *R. gnavus* were cultured in an anaerobic chamber (gas mixture 80% N_2_, 10% CO_2_ and 10% H_2_, GP[concept], Jacomex, France) at 37 °C. The bacterial cells were collected by centrifugation (10 000 rpm, 10 min, 20 °C) and each pellet was resuspended in anaerobic phosphate buffered saline (PBS, 1 mg/mL cysteine HCl, pH 6) whereby the number of colony-forming units (CFU)/mL was determined by counting the colonies on the plates of a tenfold serial dilution of the suspension before mixing together. Table 1 is showing the final concentration of each strain after mixing the original cultures.

**Table 1:** **Composition of the bacterial cocktail for oral inoculation**

Bacterial strain	Day 19 (CFU/mL)	Day 20 (CFU/mL)	Day 21 (CFU/mL)
*E. coli*	2.11 × 10^9^	1.22 × 10^9^	2.28 × 10^9^
*Enterococcus faecalis*	3.44 × 10^9^	2.28 × 10^10^	3.56 × 10^9^
*Lactobacillus salivarius*	4.78 × 10^7^	1.16 × 10^7^	2.39 × 10^7^
*Lactobacillus crispatus*	1.89 × 10^9^	7.78 × 10^7^	7.22 × 10^6^
*Clostridium perfringens*	1 × 10^7^	1.06 × 10^7^	2.78 × 10^8^
*Ruminococcus gnavus*	2.89 × 10^8^	2.78 × 10^8^	3.17 × 10^8^

Broilers in the challenge group were orally inoculated with 1 mL of a bacterial cocktail consisting of *Escherichia coli*, *Enterococcus faecalis*, *Lactobacillus salivarius*, *Lactobacillus crispatus*, *Clostridium perfringens* (netB-) and *Ruminococcus gnavus* on day 19, 20 and 21, with number of colony-forming units (CFU) per strain as indicated in the table.

### Study design

**Figure 1:**
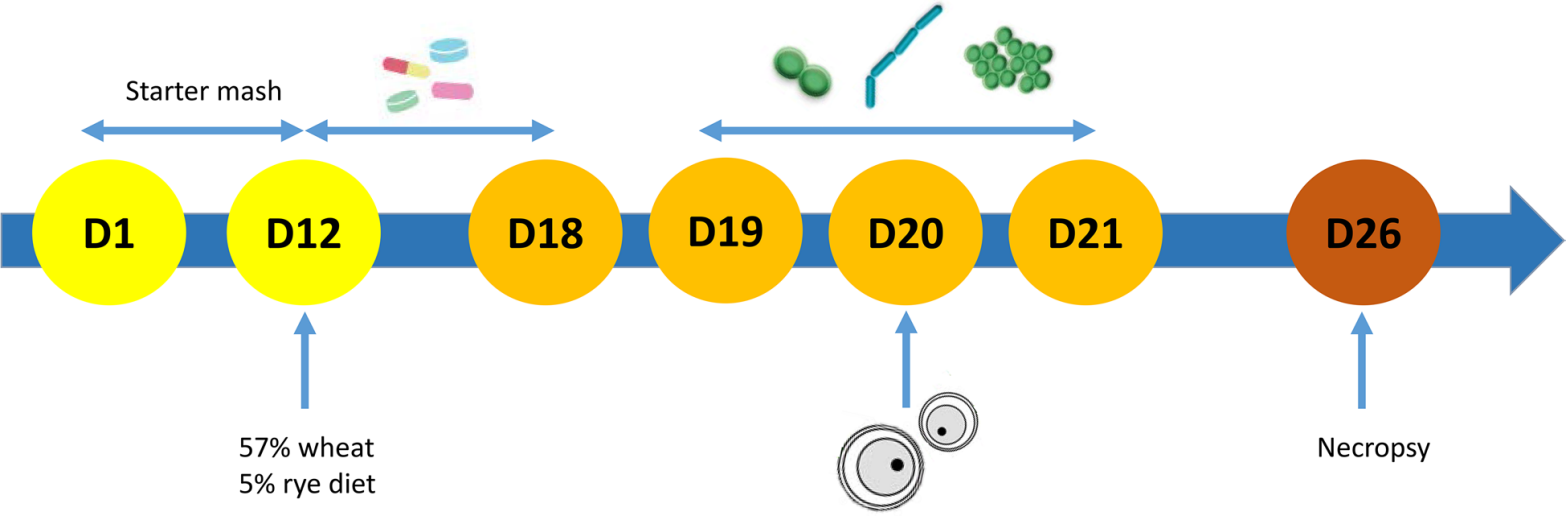
**Schematic overview of the protocol of the in vivo challenge model.** A total of 360 day-old broiler chicks (Ross 308) were randomly assigned to two groups, a control and a challenge group (9 pens per treatment and 20 broilers per pen). All animals were fed a commercial feed and switched to a wheat (57.5%) based diet supplemented with 5% rye on day 12. From day 12 to 18, all animals from the challenge group received daily 10 mg florfenicol and 10 mg enrofloxacin per kg body weight via the drinking water. After the antibiotic treatment, 1 mL of a bacterial cocktail consisting of *E. coli*, *E. faecalis*, *L. salivarius* and *L. crispatus*, *C. perfringens* (netB-) and *R. gnavus* was given daily by oral gavage from day 19 till 21. On day 20, the animals were administered a coccidial suspension consisting of 60 000 oocysts of *E. acervulina* and 30 000 oocysts of *E. maxima.* At day 26, the birds were weighed and necropsy was performed on 3 birds per pen. The duodenal loop was sampled for histological examination and content from ileum and colon was collected for protein extraction.

A total of 360 day-old broiler chicks (Ross 308) was obtained from a local hatchery and housed in floor pens on wood shavings. Throughout the study, feed and drinking water were provided ad libitum. The broilers were randomly assigned to two groups, a control and challenge group (9 pens per treatment and 20 birds per pen). All animals were fed a commercial feed till day 12 when the feed was switched to a wheat (57.5%) based diet supplemented with 5% rye (Table 2). From day 12 to 18, all animals from the challenge group received 10 mg florfenicol and 10 mg enrofloxacin per kg body weight via the drinking water daily. After the antibiotic treatment, 1 mL of the bacterial cocktail consisting of *Escherichia coli* (G.78.71), *Enterococcus faecalis* (G.78.62), *Lactobacillus salivarius* (LMG22873), *Lactobacillus crispatus* (LMG49479), *Clostridium perfringens* (netB-) (D.39.61) and *Ruminococcus gnavus* (LMG27713) was given daily by oral gavage from day 19 till 21. On day 20, the animals were administered 1 mL of a coccidial suspension consisting of 60 000 oocysts of *Eimeria acervulina* and 30 000 oocysts of *Eimeria maxima* via oral gavage. At day 26, 3 birds per pen were euthanized. The duodenal loop was sampled for histological examination and ileal and colonic content was collected and stored at − 20 °C until required for protein extraction. At day 12 and day 26, birds and feed were weighed in order to determine daily weight gain (DWG), daily feed intake (DFI) and feed conversion ratio (FCR) (Table 3). A schematic presentation of the protocol is shown in Figure 1.

**Table 2:** **Composition and nutrient content of the wheat/rye based broiler diet**

Feedstuff	Starter	Grower	Calculated nutrient composition	Starter	Grower
	%	%		%	%
Wheat	55.13	57.87	Dry matter	88.45	88.38
Rye	0.00	5.00	Ash	5.11	4.79
Soy meal, crude fiber content < 50	22.86	22.86	Crude protein	20.85	18.98
Full fat soy beans	7.50	2.50	Crude fat	10.83	9.90
Rapeseed meal < 380	2.74	0.00	Crude fiber	2.93	2.49
Animal fat	7.20	7.20	Carbohydrates	48.46	51.95
Soybean oil	1.00	1.00	Starch	34.62	38.46
Premix (maize)	0.50	0.50	Sugars	4.77	4.53
Lime fine	1.11	1.11	NDF	10.52	9.93
Monocalcium phosphate	0.83	0.83	ADF	4.18	3.52
Salt	0.18	0.18	Calcium	0.69	0.66
NaHCO_3_	0.25	0.25	Phosphorus, total	0.57	0.54
l-Lysine HCl	0.30	0.30	Calcium/dP poultry	0.22	0.22
dl-Methionine	0.30	0.30	Magnesium	0.16	0.14
l-Threonine	0.10	0.10	Potassium	0.88	0.79
			Sodium	0.15	0.15
			Chloride	0.20	0.20
			Base-excess (mEq/kg)	23.39	20.97
			Linolic acid	2.38	1.92

Starter diet, a commercial feed, administered to all broilers till day 11. From day 12 to 26, all animals received a grower diet, wheat (57.5%) supplemented with rye (5%).

**Table 3:** **Mean ± standard deviation of performance parameters**

Time period	Parameters	Control	Challenge	*p*-value
		Mean ± SD	Mean ± SD	
D1–D12	BW D12 (g)	290 ± 13	295 ± 11	0.485
	DWG (g)	19.6 ± 1	20.2 ± 1	0.342
	DFI (g)	24 ± 1	25 ± 1	0.614
	FCR	1.25 ± 0.06	1.23 ± 0.05	0.321
D12–D26	BW D26 (g)	1375 ± 58	1187 ± 46	*<* *0.001*
	DWG (g)	78 ± 4	64 ± 4	*<* *0.001*
	DFI (g)	116 ± 6	108 ± 7	*0.014*
	FCR	1.50 ± 0.07	1.69 ± 0.18	*0.004*

Body weight (BW), daily weight gain (DWG), daily feed intake (DFI) and feed conversion ratio (FCR) was measured at day 12 and day 26 for the control and challenge group. Significant differences (*p* < 0.05) are shown in italic.

### Macroscopic gut wall appearance scoring system

The macroscopic appearance of the gut was evaluated using a previously described scoring system [17], in which 10 parameters were evaluated and assigned 0 (absent) or 1 (present), resulting in a total score between 0 and 10. A total score of 0 to 2 represents a normal appearance of the intestinal tract while score 10 points to severe deviations from the normal appearance. The parameters are (1) “ballooning” of the gut; (2) inflammation, cranial to Meckel’s diverticulum; (3) macroscopically visible and tangible fragile small intestine cranial to Meckel’s diverticulum; (4) loss of tonus at longitudinal cutting of the intestine cranial to the Meckel’s diverticulum within 3 s after incision; (5) abnormal appearance of the intestinal content (excess mucus, orange content, gas) cranial to Meckel’s diverticulum; (6, 7, 8, 9) are identical to (2, 3, 4, 5) but caudal to Meckel’s diverticulum and (10) presence of undigested particles in the colon.

A coccidiosis scoring was performed as described by Johnson and Reid [24]. The animals were given a score for typical lesions associated with *E. acervulina*, *E. maxima* and *E. tenell*a. For each species, a score was given between 0 (absent) and 4 (severe). A total coccidiosis score was calculated as the sum of the three individual *Eimeria* species scores.

### Morphological parameters

The duodenal loops were fixated in 4% formaldehyde for 24 h, dehydrated in xylene and embedded in paraffin. Sections of 4 µm were cut using a microtome (HM360, Thermo Scientific, Waltham, MA, USA) and processed as described by De Maesschalck et al. [25]. After staining with haematoxylin and eosin, morphological parameters were assessed using standard light microscopy. Villus length, measured from the crypt–villus junction to the villus tip, and crypt depth, measured from the junction to the base, in the duodenum were determined by random measurement of 12 villi per section using a Leica DM LB2 microscope equipped with a camera and a computer based image analysis program, LAS V4.1 (Leica Application Suite V4, Wetzlar, Germany). The average villus length, crypt depth and villus-to-crypt ratio was determined for 3 animals per pen for 9 pens per treatment.

### Immunohistochemical examination

Antigen retrieval was performed on 4 µm duodenal sections with a pressure cooker in citrate buffer (10 mM, pH 6). Slides were rinsed with washing buffer (Dako kit, K4011, Glostrup, Denmark) and blocked with peroxidase reagent (Dako, S2023) for 5 min. Slides were rinsed with distilled water and Dako washing buffer before incubation with anti-CD_3_ primary antibodies (Dako CD_3_, A0452) for 30 min at room temperature diluted 1:100 in antibody diluent (Dako, S3022). After rinsing again with washing buffer, slides were incubated with labelled polymer-HRP anti-rabbit (Envision^+^ System-HRP, K4011) for 30 min at room temperature. Before adding di-amino-benzidine (DAB^+^) substrate and DAB^+^ chromogen (Dako kit, K4011) for 5 min, slides were rinsed 2 times with washing buffer. To stop the staining, the slides were rinsed with distilled water, dehydrated using the Shandon Varistain-Gemini Automated Slide Stainer and counterstained with hematoxylin for 10 s. The slides were analyzed with a Leica DM LB2 microscope and a computer based image analysis program LAS V4.1 (Leica Application Suite V4, Germany) to measure CD_3_ positive cells in a total area of 3.5 ± 0.5 mm^2^ which represents the area of approximately 10 to 12 villi.

### Discovery proteomics

#### Sample preparation

Five hundred mg content of each colon and ileum sample, from 1 bird per pen for 9 pens per treatment, was solubilized in 10 mL extraction buffer (2 M urea, 50 mM ammonium bicarbonate). After homogenizing by vortexing and centrifugation (20 000 × *g*, 15 min, 4 °C), the supernatant was filtered through a 0.22 µm filter (Merck, Darmstadt, Germany) directly in a Vivaspin 20 with a 5 kDa MWCO filter (Sartorius, Göttingen, Germany) and centrifuged for 1 h at 4000 × *g*. The filter was washed 3 times with 1 mL extraction buffer followed by centrifugation (4000 × *g*, 10 min, 4 °C). The samples were washed 3 times with 1 mL 500 mM triethylammonium bicarbonate buffer (TEABC, Sigma, Belgium) to remove the urea. Subsequently, the samples were concentrated to a volume of ± 500 µL. To determine the protein concentration, a Bradford assay was performed where OD was measured at 595 nm. Samples were diluted with TEABC buffer to obtain 50 µg of protein which was then reduced with dithiothreitol (DTT) from a 10 mM stock to a final concentration of 1 mM and incubated at 60 °C for 30 min, followed by alkylation for 10 min at room temperature with 200 mM methyl methanethiosulfonate (MMTS) to achieve a final concentration of 10 mM MMTS. Hereafter, 10 mM calcium chloride and 100% acetonitrile were added to a final concentration of 1 mM and 5% respectively. Finally, trypsin of a 1 µg/µL stock was added in a 1:20 (trypsin:protein) ratio for overnight digestion at 37 °C. The samples were vacuum dried and analyzed with high performance liquid chromatography–mass spectrometry (HPLC–MS).

#### HPLC–MS

Peptides were dissolved in 0.1% formic acid in HPLC-grade water (buffer A) to a final concentration of 1 µg/µL. 100 fmol of mass prep digestion standard 2 (MPDS 2) was spiked into each sample. Data Dependent Acquisition MS analysis was performed on a TripleTOF 5600 (Sciex, Darmstadt, Germany) fitted with a DuoSpray ion source in positive ion mode, coupled to an Eksigent NanoLC 400 HPLC system (Sciex). Peptides were separated on a microLC YMC Triart C18 column (id 300 μm, length 15 cm, particle size 3 μm) at a flow rate of 5 μL/min by means of trap-elute injection (YMC Triart C18 guard column, id 500 μm, length 5 mm, particle size 3 μm). Elution was performed using a gradient of 4–40% buffer B (0.1% formic acid, 5% DMSO in 80% ACN) over 90 min. Ion source parameters were set to 5.5 kV for the ion spray voltage, 30 psi for the curtain gas, 13 psi for the nebulizer gas and 80 °C as temperature. For DDA, a 2.25 s instrument cycle was repeated in high sensitivity mode throughout the whole gradient, consisting of a full scan MS spectrum (300–1250 m/z) with an accumulation time of 0.2 s, followed by 20 MS/MS experiments (50–1800 m/z) with 0.2 s accumulation time each, on MS precursors with charge state 2 to 5+ exceeding a 500 cps threshold. Rolling collision energy was used as suggested by the manufacturer and former target ions were excluded for 10 s.

#### Database searching

The *.wiff files generated during LC–MS/MS analysis were imported into the Progenesis QI for Proteomics software (non-linear dynamics). The different samples were aligned based on retention time and m/z of reoccurring features to enable relative quantification. After subsequent peak picking, a merged *.mgf file was exported from the software and searched for identifications with MASCOT Daemon (Matrix Science, version 2.5.1) against a chicken database (reviewed protein database downloaded from Swissprot, January 2016) supplemented with the cRAP database (laboratory proteins and dust/contact proteins) and the internal standard. Maximum peptide mass tolerance and fragment mass tolerance were set to 10 ppm and 0.1 Da respectively. Additionally, methylthio on cysteine was set as a fixed modification and deamidation of asparagine and/or glutamine and oxidation of methionine were set as variable modifications. Enzyme specificity was set to trypsin with a maximum of one missed cleavage. The identifications were exported from MASCOT Daemon with a 1% false discovery rate (*.xml format) and imported into Progenesis QI for Proteomics.

### Statistical analysis

Statistical analysis of performance parameters, macroscopic scoring, intestinal morphology and immunohistochemistry was performed using Graphpad Prism (v.5, San Diego, USA). Statistical differences in macroscopic gut appearance and coccidiosis scores were determined using the Chi-square test. For the other parameters, pen (total of 3 birds per pen) was taken as experimental unit. To evaluate whether the data were normally distributed, a Kolmogorov–Smirnov test was performed. In case of a normal distribution, comparison between the control and challenge group was performed with an independent samples t-test. Otherwise, the non-parametric Mann–Whitney test was performed. Progenesis (v4.1) was used for analysis of the proteomics data. Overall, a *p*-value of ≤ 0.05 was considered statistically significant.

## Results

### Performance parameters

Body weight (BW), daily weight gain (DWG), daily feed intake (DFI) and feed conversion ratio (FCR) were measured at day 12 and 26. At day 26, challenged birds had a significant lower BW, DWG and DFI whereas FCR was increased (*p* < 0.05) (Table 3).

### Macroscopic scoring

**Figure 2:**
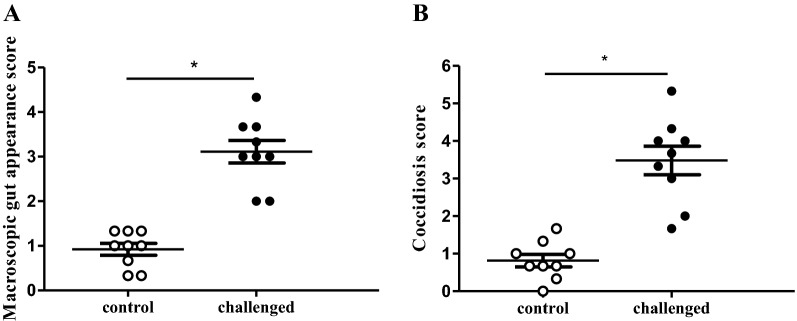
**Macroscopic scoring parameters of intestine from broilers derived from the in vivo gut damage trial.** Each dot represents the average score of 3 birds per pen with a total of 9 pens for control (white; *n* = 27) and challenged (black; *n* = 27) birds. **A** Macroscopic gut appearance score was determined by scoring of 10 parameters on assessment of absence (0) or presence (1) resulting in a total score between 0 and 10. **B** Coccidiosis score was determined by the sum of the individual species scores, 0 if absent to 4 if severe, for lesions caused by *Eimeria* (*E.*) *acervulina*, *E. maxima* and *E. tenella*. Asterisk denotes statistical significance of *p *< 0.0001 between control and challenged animals.

The scores for macroscopic gut wall appearance (3.11 ± 0.76 vs 0.92 ± 0.4) and coccidiosis (3.48 ± 1.14 vs 0.82 ± 0.5) were higher in the challenge group compared to the control group respectively (*p *< 0.0001) (Figure 2).

### Intestinal morphology and immunohistochemistry

**Figure 3:**
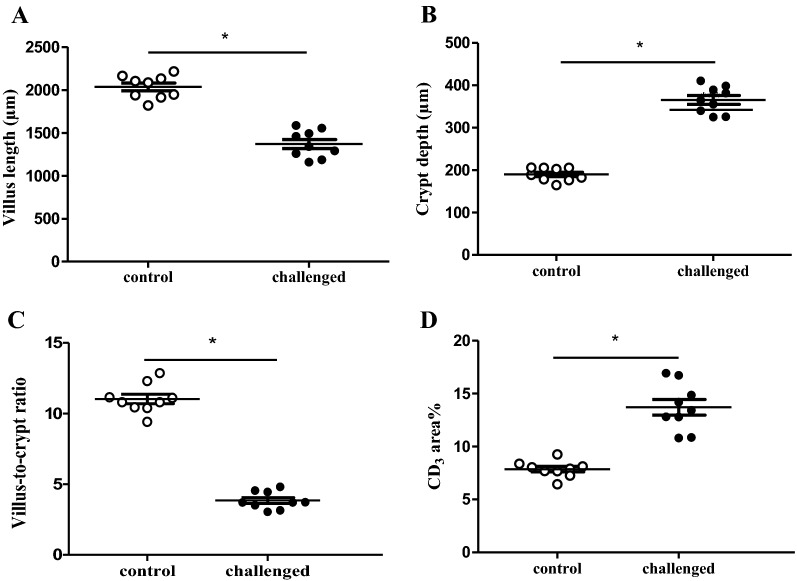
**Histological parameters in duodenal sections from birds derived from the in vivo gut damage trial.** 12 villi were randomly selected and measured per section and per animal using Leica DM LB2 microscope and a computer based image analysis program, LAS V4.1. Each dot represents the mean of 3 birds per pen with a total of 9 pens for control (white; *n* = 27) and challenged (black; *n* = 27) birds. **A** Villus length was measured from the crypt–villus junction to villus tip. **B** Crypt depth was measured from the junction to the base. **C** Villus-to-crypt ratio. **D** T-lymphocyte infiltration (CD_3_ area%) was measured for a total area of 3.5 ± 0.5 mm^2^. Asterisk denotes statistical significance of *p* < 0.0001 between control and challenge group.

Significantly shorter villi (1370 ± 158.7 µm vs 2036 ± 134.6 µm), deeper crypts (365.7 ± 31.4 µm vs 190.1 ± 15.4 µm), a lower villus-to-crypt ratio (3.85 ± 0.63 vs 11.03 ± 1.03) and higher infiltration of CD_3_^+^ cells (13.71 ± 2.22 area% vs 7.86 ± 0.77 area%) (*p *< 0.0001) were detected in the duodenal sections of animals from the challenge group as compared to the control group respectively (Figure 3).

### Discovery proteomics

**Figure 4:**
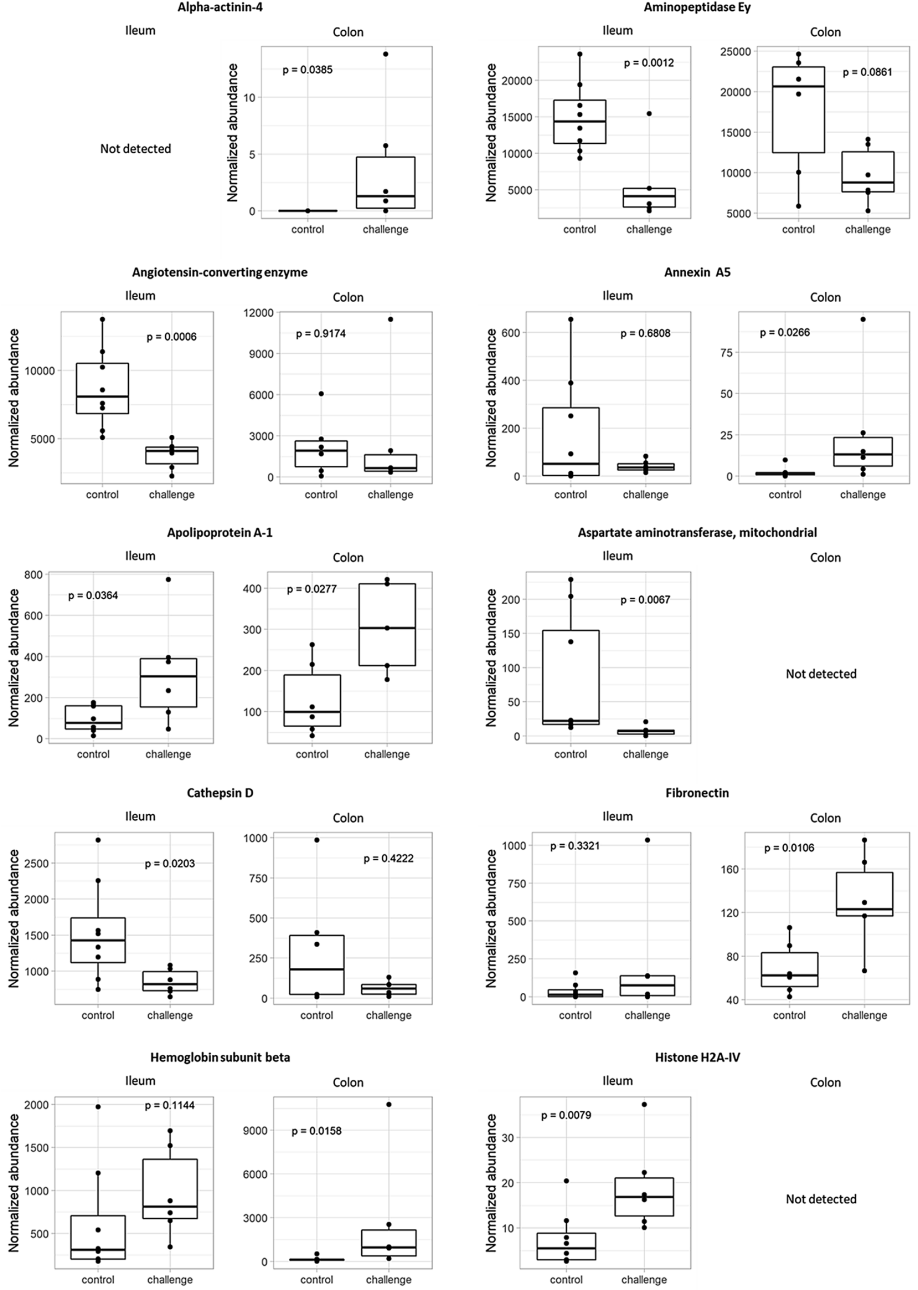

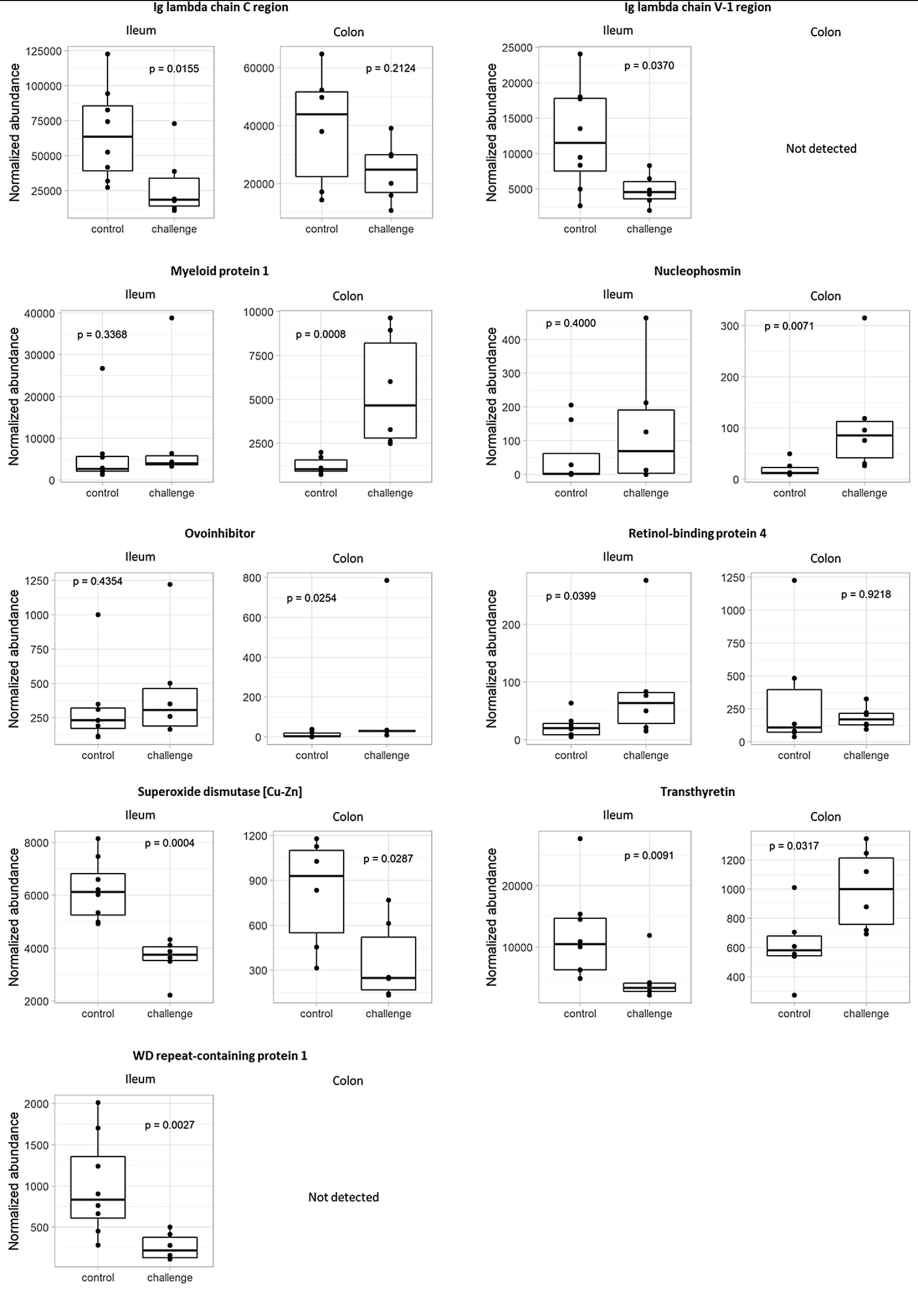
**Protein abundances in ileal and colonic content of birds from the in vivo gut damage trial.** Proteomic analysis using high performance liquid chromatography–mass spectrometry (HPLC–MS) resulted in detection of proteins with a significant difference between groups in normalized abundance (*p *< 0.05) in ileal and/or colonic content. Each dot represents 1 bird per pen with a total of 9 pens for control (white; *n* = 9) and challenged (black; *n* = 9) animals.

Using MASCOT Daemon (Matrix Science, version 2.5.1) against a chicken database (reviewed protein database downloaded from Swissprot, January 2016) supplemented with the cRAP database (laboratory proteins and dust/contact proteins), 157 and 181 proteins were identified for colon and ileum respectively. In broilers from the challenge group, the following proteins showed a significantly higher abundance compared to control animals in colonic content (*p* < 0.05): alpha-actinin-4 (ACTN4), annexin A5 (ANXA5), apolipoprotein A-1 (APOA1), fibronectin (FINC), hemoglobin subunit beta (HBB), myeloid protein 1 (MIM1), nucleophosmin (NPM), ovoinhibitor (IOV7) and transthyretin (TTR). Both in colonic and ileal content, superoxide dismutase [Cu–Zn] (SOD) showed a decreased abundance compared to control animals (*p *< 0.05). Angiotensin-converting enzyme (ACE), mitochondrial aspartate aminotransferase (AATM), cathepsin D (CATD), Ig lambda chain C region (LAC), Ig lambda chain V-1 region (LV1), TTR and WD repeat-containing protein 1 (WDR1) showed a lower abundance in challenged birds (*p *< 0.05) in ileal samples. Following proteins were more abundant in challenged birds (*p *< 0.05): APOA1, histone H2A-IV (H2A4) and retinol-binding protein 4 (RET4) (Figure 4, Tables 4 and 5).

**Table 4:** **Proteins of which the normalized abundance in colonic content was significantly different between control and challenged groups**

Accession number	Protein name	Abbreviation	*p*-value	Highest mean
Q90734	Alpha-actinin-4	ACTN4	0.0385	Challenge
P17153	Annexin A5	ANXA5	0.0266	Challenge
P08250	Apolipoprotein A-1	APOA1	0.0277	Challenge
P11722	Fibronectin	FINC	0.0106	Challenge
P02112	Hemoglobin subunit beta	HBB	0.0158	Challenge
P08940	Myeloid protein 1	MIM1	0.0008	Challenge
P16039	Nucleophosmin	NPM	0.0071	Challenge
P10184	Ovoinhibitor	IOV7	0.0254	Challenge
P80566	Superoxide dismutase [Cu–Zn]	SOD	0.0287	Control
P27731	Transthyretin	TTR	0.0317	Challenge

Proteomics using high performance liquid chromatography–mass spectrometry (HPLC–MS) was performed on colonic content of animals from the control (*n* = 9) and challenged (*n* = 9) group at day 26. This resulted in 9 proteins with a significantly higher normalize abundance (*p* < 0.05) and 1 protein with a significantly lower normalized abundance (*p* < 0.05) in challenged birds.

**Table 5:** **Proteins of which the normalized abundance in ileal content was significantly different between control and challenged groups**

Accession number	Protein name	Abbreviation	*p*-value	Highest mean
O57579	Aminopeptidase Ey	AMPN	0.0012	Control
Q10751	Angiotensin-converting enzyme	ACE	0.0006	Control
P08250	Apolipoprotein A-1	APOA1	0.0364	Challenge
P00508	Aspartate aminotransferase, mitochondrial	AATM	0.0067	Control
Q05744	Cathepsin D	CATD	0.0203	Control
P02263	Histone H2A-IV	H2A4	0.0079	Challenge
P20763	Ig lambda chain C region	LAC	0.0155	Control
P04210	Ig lambda chain V-1 region	LV1	0.0370	Control
P41263	Retinol-binding protein 4	RET4	0.0399	Challenge
P80566	Superoxide dismutase [Cu–Zn]	SOD	0.0004	Control
P27731	Transthyretin	TTR	0.0091	Control
O93277	WD repeat-containing protein 1	WDR1	0.0027	Control

Proteomics using high performance liquid chromatography–mass spectrometry (HPLC–MS) was performed on ileal content of animals from the control (*n* = 9) and challenge (*n* = 9) group at day 26. This resulted in 3 proteins with a significantly higher normalized abundance (*p* < 0.05) and 9 proteins with a significantly lower normalized abundance (*p *< 0.05) in challenged birds.

## Discussion

Intestinal inflammation models that do not induce clinical signs, but affect zootechnical parameters by using triggers that are common under field conditions are the models of choice to identify biomarkers for intestinal health. Coccidiosis is the most common intestinal disease in broilers, and coccidiosis lesions caused by *E. maxima* and *E. acervulina* are frequently observed in broilers [26, 27]. The diet shift, antibiotic treatment, and subsequent oral administration of a mix of bacterial opportunistic pathogens and commensals, was performed to mimic a decrease in bacterial diversity and to induce dysbiosis. Feed changes to more non-starch polysaccharide (NSP) containing cereals (e.g. rye) have been shown to affect microbial composition and animal performance [10, 28]. *E. coli* and *R. gnavus* are enriched in human patients with inflammatory bowel disease (IBD) often characterized with increased disease activity [29]. Also excess accumulation of lactic acid bacteria has been described to form part of a dysbiotic microbiota in humans, although these can also be beneficial, but this seems to be species and even strain dependent [30]. In addition, intestinal overgrowth of *C. perfringens*, even netB negative strains which are considered to be non-pathogenic for poultry, in combination with other predisposing factors such as *Eimeria* and indigestible NSPs are considered to induce small intestinal microbial perturbations [31]. The triggers used in the current model are thus biologically relevant, in contrast to chemical triggers of inflammation, such as dextran sulphate sodium (DSS), lipopolysaccharide (LPS) or feed restriction models that have been used in other studies in broilers [21, 32].

The model used in the current study led to shorter villi, deeper crypts and more infiltration of CD_3_^+^ T-cells, in association with performance losses, but without clinical symptoms. These findings confirm that the in vivo model can be used to evaluate gut damage and to identify gut health biomarkers. Detection of protein biomarkers was performed in ileal and colonic content. The latter was chosen as a proxy for fecal material, to exclude issues that are related to protein breakdown in litter and lack of homogeneity of mixed fecal material, what are likely main problems in practical development of protein quantification methods in the field.

In the current study, biomarker candidates were selected based on significant differences (*p* < 0.05) in protein abundance between control and challenged birds in ileal and/or colonic content. The normalized protein abundance mostly was higher in the ileal content compared to the colonic content samples (Figure 4), which indicates that the used model induces proximal intestinal damage following release of host proteins in ileal content, and degradation of these proteins during passage further down the intestinal tract occurs.

The proteins that were identified as more abundant in challenged animals compared to control animals were associated with inflammation, serum leakage and tissue destruction. A clear inflammation marker that was identified as more abundant in the colon of challenged animals was myeloid protein 1 (MIM1) and was the most discriminating protein detected between control and challenged birds (Table 4). Myeloid protein 1 is expressed in myeloid cells and is present in large amounts in secretory granules of heterophilic granulocytes [33]. Amongst other leukocytes, activated heterophils can infiltrate the mucosa, transmigrate through the intestinal epithelium and degranulate in the intestinal lumen when an inflammatory trigger targets the epithelium [34, 35]. High fecal or intestinal content levels of MIM1 are thus specific for inflammatory intestinal disorders. In man, a neutrophil granule protein, calprotectin, is used in clinical practice to evaluate intestinal inflammation [36]. Fecal calprotectin levels reflect disease activity in IBD [37]. The MIM1 protein, and in extension all heterophil secretory granule proteins, may thus represent useful intestinal inflammation biomarkers.

Tissue damage markers are a second set of proteins that are more abundant in the intestinal lumen of challenged compared to control animals. Damage of the intestinal epithelial barrier, invasion by micro-organisms or entry of antigens in the mucosal tissue below the epithelial layer leads to mucosal inflammation and subsequent tissue damage [36]. Due to the tissue damage or destruction, tissue specific proteins can be released and end up in intestinal content. One of these proteins is fibronectin (FINC). Fibronectin is a high molecular weight glycoprotein that is found in the basement membrane and extracellular matrix in the intestinal wall (insoluble form) and in plasma (soluble form) [38]. It has been shown that the expression of epithelial-derived fibronectin is highly upregulated in DSS-induced colitis models [39]. Fibronectin can potentiate cell attachment and wound healing through epithelial-matrix interactions and its expression is supposed to be vital for maintaining normal epithelial integrity as well as regulating epithelial response to injury during colitis [39]. Intestinal damage in our current study could thus have resulted in an increased expression of epithelial or fibroblast-derived fibronectin, release of matrix fibronectin or even leakage of plasma fibronectin, explaining the increased levels in the intestinal content of challenged animals.

Two nuclear proteins were identified as being more abundant in intestinal content of challenged animals, namely histone H2A-IV (H2A4) and nucleophosmin (NPM). H2A4 is a core component of nucleosomes that wraps and packages DNA into chromatin [40]. The high abundance in challenged animals may be explained by increased cell death. NPM is a phosphoprotein that initiates p53, a tumor suppressor gene which drives the cell to apoptosis [41]. Overexpression of NPM is associated with cell proliferation [42]. Parker et al. [43] states that cell proliferation within small intestinal crypts is the primary force that drives cell migration along the villus. Challenged broilers had longer crypts than the control animals which indicates more cell proliferation, likely in an attempt to repair lesions.

Epithelial damage has been shown to be associated with altered protein expression and distribution of intercellular connections, including tight junctions [44]. Alpha-actinin-4 (ACTN4), found to be more abundant in intestinal content in the challenge group as compared to the control group, was shown to be localized in the apical part of chicken intestinal epithelial cells [45], more specifically as a component of the tight junction (*zonula occludens*) [46] and/or belt desmosomes (*zonula adherens*) [47]. Changes in gut permeability induced by enteric pathogens and/or parasites can also be the consequence of damage to these tight junctions [48]. Release of alpha-actinin-4 in the intestinal content of challenged animals might thus result from a higher expression and damaged tight junction and zonula adherens complexes.

Reduced tight junction integrity allows paracellular translocation of large molecules in both directions, thus loss of plasma proteins from the host into the intestinal lumen and undesirable foreign substances from the lumen into the blood. Multiple proteins found to be higher abundant in the intestinal content of challenged animals are hypothesized to leak from the serum to the intestinal content because of mucosal damage. In our current model, the dysbiosis and the coccidial infection might have resulted in production of acute-phase proteins (APPs) by the liver [49]. In mammalian species, APPs apolipoprotein A-1 (APOA1), transthyretin (TTR) and retinol-binding protein 4 (RET4) behave as negative APPs of which the concentration decreases in plasma during an acute phase response (APR). The exact behavior of the expression in the liver of chickens is unknown [50]. Apolipoprotein A-1 (APOA1) is the major protein fraction of high-density lipoprotein (HDL) particles in plasma [51]. However, in avian species, APOA1 could also be expressed in numerous tissues other than the liver, such as absorptive intestinal epithelial cells, so our data could imply intestinal epithelial cell loss [52, 53]. Transthyretin (TTR) is a highly conserved protein in animal species that is involved in transport of thyroid hormones and retinol bound to retinol-binding protein 4 (RET4) in the bloodstream [54]. Retinol (vitamin A) is known to be essential for differentiation and proliferation of epithelial cells [55]. Ovoinhibitor (IOV7) is a serine proteinase inhibitor that can reduce enzymatic digestion by trypsin and chymotrypsin [56]. The detection of hemoglobin subunit beta (HBB) in intestinal content indicates that the administered challenges induced gut leakage and likely also endothelial damage allowing red blood cell leakage from the blood to the lumen.

A final protein that was found to be more abundant in colonic content of challenged animals is annexin A5 (ANXA5). Both apoptotic and necrotic cells expose phosphatidylserine (PS), a major “eat-me” signal for phagocytes and for which ANXA5 binds with high affinity and specificity followed by initiation of an immune reaction [57]. Van Genderen [58] suggests that the presence of extracellular ANXA5 is a consequence of release of cytosolic content from dead cells into the surrounding environment.

Proteins that were identified as less abundant in challenged animals compared to control animals were epithelial cell activity markers and antibody components. A variety of peptidases, such as aminopeptidase Ey (AMPN) and angiotensin-converting enzyme (ACE), are localized in the intestinal brush border membrane and are involved as major functional enzymes in the final stage of protein digestion in the small intestine [59]. In case of bacterial infection, epithelial cells could be damaged with loss of the functional brush border membrane. In rats, the distribution of ACE activity is highest in the proximal to middle region of the intestine, decreasing distally [59]. In our study, AMPN was annotated by more than 65 unique peptides, both in ileum and colon, while the average unique peptide level per protein was 6. This indicates a high chance of detectability and stability of AMPN in intestinal content. It seems plausible that lower concentrations of brush border enzymes are found when less epithelial cells are present, thus in the intestinal content of challenged animals.

It is probable that following proteins are linked to highly metabolically active cells which results in a reduced abundance in case of gut health problems. Superoxide dismutase [Cu–Zn] (SOD) catalyzes the dismutation of superoxide radicals to hydrogen peroxide (H_2_O_2_) and oxygen and contributes to enhanced small intestinal preservation in animals [60]. Superoxide dismutases consist of three isoforms in mammals, namely the cytoplasmic, mitochondrial and extracellular Cu–Zn SOD [61]. Chicken SOD has 71.2%, 14.8% and 24.4% identity respectively with the human isoforms suggesting that in our study the cytoplasmic isoform of chicken SOD was identified. Mitochondrial aspartate aminotransferase (AATM), formerly known as glutamic-oxaloacetic transaminase, catalyzes the reaction of l-aspartate and 2-oxoglutarate to oxaloacetate and glutamate [62]. WD (tryptophan-aspartate) repeat-containing protein 1 (WDR1), also called actin-interacting protein 1 (AIP1), acts as a cofactor of ADF-cofilin and facilitates actin turnover by disassembly of actin filaments [63]. Lechuga [64] suggests that this protein is necessary for intestinal epithelial morphogenesis due to its abundance at epithelial apical junctions which was substantiated with the observation that downregulation of AIP1 expression increased paracellular permeability and reduced junctional recruitment of adherens and tight junction proteins. This is consistent with our data, showing a reduced abundance in the ileum of challenged birds. Cathepsin D (CATD), an aspartic proteinase expressed in lysosomes, is the second most abundant protease after pepsin in the chicken gastrointestinal tract [65], thus suggesting that, in chicken, CATD may play additional roles, other than just being a “housekeeping enzyme” necessary for autophagy [66]. It could play a role as a digestive enzyme, as has been suggested in fish [67].

Antibody light chain proteins were found less abundant in the ileal content of challenged animals compared to control animals. In birds, immunoglobulins IgA, IgM and IgY (also named chicken IgG) are antibodies produced by B-cells as a response to presented antigens and consist of two heavy and light chains whereby birds only have one isotope of light chain, namely lambda (λ). The light chain is made up of a constant, Ig lambda chain C region (LAC), and a variable region, Ig lambda chain V-1 region (LV1) [68]. IgAs are the most predominant class of antibodies in mucosal secretions with their primary function to maintain homeostasis at the mucosal surface and thus a steady-state condition in the gut [69]. However, the reason for lower abundance in challenged birds is not clear.

In conclusion, using a broiler gut inflammation model, we have identified candidate intestinal biomarkers in ileal and/or colonic content of which concentrations differed significantly between control and challenged animals. Ideal potential biomarkers should be highly discriminative between challenged and control groups, and have high but no overlapping numerical values between groups, to have a good chance of being applicable in the field. Since the model was developed using triggers that are common in field conditions, these potential biomarkers can serve as basis for validation in the field via development of easy and rapid diagnostic tools as a method to detect and measure gut barrier failure in broilers.

## Abbreviations

AATM: mitochondrial aspartate aminotransferase
ACE: angiotensin-converting enzyme
ACTN4: alpha-actinin-4
ANXA5: annexin A5
APOA1: apolipoprotein A-1
BHI: brain heart infusion
BW: body weight
CATD: cathepsin D
CFU: colony-forming unit
DAB: di-amino-benzidine
DDA: data dependent acquisition
DFI: daily feed intake
DSS: dextran sulphate sodium
DTT: dithiothreitol
DWG: daily weight gain
FCR: feed conversion ratio
FINC: fibronectin
H2A4: histone H2A-IV
HBB: hemoglobin subunit beta
HPLC-MS: high performance liquid chromatography–mass spectrometry
IBD: inflammatory bowel disease
IOV7: ovoinhibitor
LAC: Ig lambda chain C region
LB: Luria–Bertani
LPS: lipopolysaccharide
LV1: Ig lambda chain V-1 region
MIM1: myeloid protein 1
MMTS: methanethiosulfonate
MRS: Man–Rogosa–Sharpe
NPM: nucleophosmin
NSP: non-starch polysaccharides
PBS: phosphate buffered saline
RET4: retinol-binding protein 4
SOD: superoxide dismutase [Cu–Zn]
TTR: transthyretin
WDR1: WD repeat-containing protein 1
